# Clearing-House for Unmarried Mothers

**Published:** 1920-10-23

**Authors:** 


### Clearing-House for Unmarried Mothers.

Circularising local authorities, the National Council for the Unmarried Mother and her Child states that, in view of the powers by which local authorities are able to make provision for necessitous unmarried mothers with their children, it is prepared to co-operate on the following lines: (1) The Council will act as a clearing-house for accommodation for the unmarried mothers and their children; (2) it will supply literature touching the problem of illegitimacy; (3) it will advise on equipment and maintenance of hostels; (4) it will bring suitable foster-mothers to the notice of local authorities; (5) it is prepared to send speakers with special experience to conduct propaganda work and, as special work, to undertake the survey of a neighbourhood in order to find and register suitable foster-mothers under provisions for the unmarried mother and her child.

Some of the London Borough Councils have already decided to support the scheme by making donations to the National Council.

